# RIPK2-Mediated Autophagy and Negatively Regulated ROS-NLRP3 Inflammasome Signaling in GMCs Stimulated with High Glucose

**DOI:** 10.1155/2019/6207563

**Published:** 2019-08-14

**Authors:** Chenlin Gao, Jiao Chen, Fang Fan, Yang Long, Shi Tang, Chunxia Jiang, Jiying Wang, Youhua Xu, Yong Xu

**Affiliations:** ^1^Endocrinology Department, The Affiliated Hospital of Southwest Medical University, Sichuan Luzhou 646000, China; ^2^State Key Laboratory of Quality Research in Chinese Medicine, Faculty of Chinese Medicine, Macau University of Science and Technology, Avenida Wai Long, Taipa, Macau, China; ^3^Department of Endocrinology, The Third Hospital of Mianyang, Sichuan 621000, China; ^4^Department of Endocrinology, The First People's Hospital of Neijiang, Sichuan 641000, China; ^5^Luzhou Key Laboratory of Cardiovascular and Metabolic Diseases, Affiliated Hospital of Southwest Medical University, Luzhou, Sichuan 646000, China; ^6^Endocrinology Department, The Affiliated Hospital of Nuclear Industry 416 Hospital, Chengdu, Sichuan 610000, China; ^7^Department of Endocrinology, Guangyuan Central Hospital, Guangyuan, Sichuan 628000, China

## Abstract

**Background:**

Hyperglycemia plays a vital role in diabetic nephropathy (DN); autophagy and its potential upregulator receptor-interacting protein kinase 2 (RIPK2) are associated with ROS, which play a potential role in regulating NLRP3, and may be involved in inflammation in DN.

**Aim:**

In this study, we aimed to explore the mechanisms mediated by RIPK2 in autophagy and the relationship with ROS-NLRP3 of DN, by investigating the levels of RIPK2 and autophagy in glomerular mesangial cells (GMCs) stimulated with high glucose.

**Material and Methods:**

GMCs were divided into the following groups: normal group (NC), high glucose group (HG), and RIPK2 siRNA group. RIPK2, LC3, caspase1, and IL-1*β* levels were measured by western blotting and RT-PCR. Autophagosomes were measured by GFP-RFP-LC3; ROS were detected by DCFH-DA.

**Results:**

High glucose upregulated RIPK2 and LC3 in GMCs during short periods (0-12 h) (*p* < 0.01), while RIPK2 and LC3 were significantly downregulated in the long term (12-72 h) (*p* < 0.01); these changes were positively correlated with glucose concentration (*p* < 0.01). In addition, levels of ROS, caspase1, and IL-1*β* increased in a time- and dose-dependent manner in the high glucose group, even with an increased expression of LC3 (*p* < 0.01). However, LC3 expression decreased in the siRIPK2 group, while levels of ROS, caspase1, and IL-1*β* increased (*p* < 0.01).

**Conclusions:**

Autophagy was activated by high glucose at short time periods but was inhibited in the long term, demonstrating a dual role for high glucose in autophagy of GMCs. RIPK2 regulates ROS-NLRP3 inflammasome signaling through autophagy and may be involved in the pathogenesis of DN.

## 1. Introduction

Diabetes is one of the main public health problems, and its prevalence has been increasing significantly in recent years; in 2017, the International Diabetes Federation (IDF) reported 425 million adult patients with diabetes worldwide, and over 350 million people are at risk of diabetes [1]. Diabetic nephropathy (DN) is one of the main microvascular complications of diabetes and has become the most common cause of end-stage renal disease in developed countries [2]. However, the etiology and pathogenesis of DN are complex; numerous studies have shown that oxidative stress and inflammation play a central role in the pathological process in DN, which has also been confirmed in our previous experiments [3].

Autophagy clears damaged proteins and organelles to maintain cellular homeostasis [4]. The role of autophagy has been investigated in many types of kidney disease models, such as acute kidney injury, age-related kidney disease, DN, and polycystic kidneys [5–8]. However, the mechanism of autophagy in diabetic renal damage is still unclear; most studies have confirmed that autophagy activity is suppressed in the kidneys of diabetic mice [7, 9, 10], and excessive activation of autophagy may aggravate renal cell apoptosis. However, some studies have found that high glucose-induced LC3 significantly increased cell apoptosis in renal tubular cells [11]. NOD1 and NOD2 are members of the NOD-like receptor (NLR) family of intracellular pattern-recognition molecules (PRMs), which recognize fragments of bacterial peptidoglycan and initiate innate immune responses; the receptor-interacting protein kinase 2 (RIPK2) is the best-studied interaction partner of NOD and is important for activating NF-*κ*B- or MAPK-dependent inflammatory responses [12–16]. NOD1/NOD2-RIPK2 signaling is a critical pathway that links renal injury to inflammation in DN, which was demonstrated in our previous study [17]. In addition, recent reports have demonstrated that NOD1/NOD2 stimulates autophagy as an antibacterial response [18–20], while RIPK2 may play a dual role in NOD1/NOD2-dependent autophagy [21].

High glucose induces oxidative stress, and accumulation of damaged mitochondria and increased ROS production in mesangial cells can induce autophagy to remove damaged mitochondria and recycle energy. Nevertheless, autophagy may play a dual role in pathological states, for example, excessive autophagy activated by mitochondrial damage and oxidative stress in response to high glucose results in autophagic cell damage, thus aggravating cell apoptosis [22–24]. So far, the role of autophagy in high glucose-induced inflammation in mesangial cells is controversial. We therefore designed these investigations based on the following hypotheses: (i) autophagy negatively regulates high glucose-induced ROS-NLRP3 inflammasome signaling; (ii) high glucose plays a dual role in RIPK2-mediated autophagy in GMCs.

## 2. Materials and Methods

### 2.1. Cell Culture and Experimental Groups

Primary cultures of mouse glomerular mesangial cells (SV cells) were purchased from the Chinese Academy of Sciences Cell Bank and grown as monolayers at a density of 5 × 10^4^ cells/cm^2^ in low glucose DMEM (Hyclone, USA) with 10% fetal bovine serum (Bovogen, Australia) and 100 units/mL penicillin and 100 *μ*g/mL streptomycin (Beyotime, China); cells were incubated at 37°C in humidified air containing 5% CO_2_. At day 1 after seeding, the cultured cells were randomly divided into the following five groups and treatments: NC group (low glucose, 5.6 mmol/L), high glucose group (HG1, 10 mmol/L glucose; HG2, 20 mmol/L glucose; and HG3, 30 mmol/L glucose), OP group (5.6 mmol/L glucose+22.4 mmol/L mannitol), NC+siRIP2 group, HG3+siRIP2 group, NC+rapamycin (10 nmol/L rapamycin) group, and HG3+rapamycin group (10 nmol/L rapamycin). These were then further subdivided as follows: the HG3 group was divided into seven groups according to the simulation time of HG3, including 0 h, 2 h, 6 h, 12 h, 24 h, 48 h, and 72 h. The second experiment involved subdividing into five groups according to the concentration of HG, comprising the NC group, HG1 group, HG2 group, HG3 group, and the OP group; each group was cultured for 12 h and 72 h. The third experiment involved subdividing into four groups, including the NC group, NC+rapamycin group, HG3 group, and HG3+rapamycin group; each group was cultured for 72 h. The fourth experiment involved subdividing into four groups, including the NC group, NC+siRIPK2 group, HG3 group, and HG3+siRIPK2 group; each group was cultured for 12 h.

### 2.2. Transfection Assay

SVs were transfected with either siRNA-targeting RIPK2 or negative control siRNA using Lipofectamine 2000 reagent (mouse, RiboBio, Guangzhou, China) according to the manufacturer's instructions. After transfection for 48 h to inhibit RIP2 expression, transfected cells were confirmed by western blot analysis of RIPK2 protein expression; the targeted siRNA sequence for RIPK2 was 5-GGGAAGTGTTATCCAGAAA-3 (mouse, RiboBio, Guangzhou, China). By confirming the effective siRNA-targeted RIPK2, we repeated the above transfection steps; after transfection for 48 h to inhibit RIPK2 expression, cells were incubated with HG (30 mM) for 12 h; then cells, cell lysates, and total RNA were collected for further analysis.

### 2.3. Western Blotting

SVs were lysed with Radio-Immunoprecipitation Assay (RIPA) lysis and extraction buffer containing complete protease inhibitor mixture (Roche, China). The protein concentration was determined using bicinchoninic acid (BCA) analysis (BioWorld, USA). Lysates were resolved on SDS-PAGE and transferred to PVDF membranes (Millipore, USA) by electroblotting. Membranes were blocked in 5% nonfat milk and incubated overnight with primary antibody at 4°C and for 1 h with secondary HRP-tagged antibody at room temperature. Immunoblot analysis was performed using primary antibodies against RIP2 (rabbit, 62 kDa, 1 : 1000; CST, USA), LC3II (rabbit,14-16 kDa, 1 : 1000; Abcam, USA), caspase1 (rabbit, 25 kDa, 1 : 1000; Abcam, USA), IL-1*β* (rabbit, 17 kDa, 1 : 800; Abcam, USA), *β*-actin (mouse, 42 kDa, 1 : 2000; Beyotime, China), and HRP-GAPDH antibody (37 kDa, 1 : 10000; BioWorld, USA). Bands were visualized with HRP-conjugated secondary antibodies (anti-rabbit and anti-mouse, 1 : 3000, Beyotime, China). Proteins were detected using the enhanced chemiluminescence (ECL) system and ECL Hyperfilm (Millipore, USA) and quantified with Quantity One software (Bio-Rad Laboratories, Hercules, USA).

### 2.4. RT-PCR

Total RNA was extracted from SVs using an RNA extraction kit (Tiangen Biotech, China). A total of 500 ng RNA was reverse transcribed at a final volume of 20 *μ*L using a Takara RNA PCR kit (TOYOBO, Japan). cDNA (2 *μ*L) was amplified in a gradient thermal cycler using the PCR Master Mix (Ribo, China). Results were determined using a UV transilluminator and normalized to *β*-actin gene expression.

### 2.5. Confocal Microscopy

SVs were seeded in a confocal culture dish (2 cm^2^, Costar) and at a density of 1 × 10^4^ cells/cm^2^; at day 1 after seeding, cells were infected with mRFP-GFP-LC3-labeled adenovirus (Hanbio, China) at 5 MOI in different treatment culture media, the plasmid expressing a monomeric RFP-GFP-tagged LC3 (tfLC3), an autophagic flux reporter comprised of LC3 protein fused with monomeric red fluorescent protein (mRFP) and green fluorescent protein (GFP). The GFP signal was quenched within the lysosome lumen by the acidic and/or proteolytic environment. Yellow puncta in colocalized GFP (green) and mRFP (red) fluorescent signals in the cytoplasm indicate early autophagosomes, while the mRFP signals alone (red) represent late autolysosomes [25]. The LC3+ puncta numbers were examined dynamically on a Nikon C1Si laser-scanning confocal microscope in planned time. Data analysis was performed using ImageJ.

### 2.6. Reactive Oxygen Species (ROS) Evaluation

The intracellular ROS levels in SVs were evaluated using a ROS detection kit (Nanjing Jian Cheng Institute of Biotechnology, China). GMCs were grown in 24-well plates and were incubated in serum-free media containing DCFH-DA (10 *μ*M/L) in the presence of groups for 20 min at 37°C and 5% CO_2_ in the dark. After washing with PBS for three times, the conversion of DCFH-DA to the fluorescent product DCF was measured using a spectrofluorometer with excitation at 488 nm and emission at 525 nm. Background fluorescence (conversion of DCFH-DA in the absence of cells) was corrected by the inclusion of parallel blanks.

### 2.7. ELISA

Cells were plated in triplicate and were processed according to the above groups; cell supernatants were collected for IL-1*β*-specific ELISA analysis according to the manufacturer's instructions; they were normalized to the total protein in the homogenate by BCA protein assay (Pierce, USA) and expressed as cytokine concentration per milligram total protein.

### 2.8. Immunofluorescence Analysis

SVs at exponential growth state were seeded into 24-well cell culture plates and administrated with vehicle, NC group, NC+rapamycin (10 nmol/L rapamycin), HG3, and HG3+rapamycin (10 nmol/L rapamycin); each group was cultured for 72 h. After being gently washed with PBS, the cells were transferred onto the antiunloading slide and fixed with 4% paraformaldehyde. After being blocked with rabbit serum for 1 h at 37°C, the cells were incubated with primary antibodies including caspase1 (1 : 100) and IL-1*β* (1 : 100) for 24 h at 4°C followed by PBS washing for three times. FITC- or PE-conjugated secondary antibodies were applied to observe the proteins' expression under the fluorescence microscope.

### 2.9. Statistical Analysis

All experimental data were obtained from three independent experiments and shown as a representative example. Data were expressed as the mean ± SD (*x* ± *s*) and were analyzed using the SPSS 24.0 statistical package. The *p* values for differences between two groups were determined by two-tailed *t*-test; ANOVA was used for comparison of more than two groups, followed by the LSD post hoc test for multiple comparisons. A *p* value of <0.05 was defined as statistically significant.

## 3. Results

### 3.1. High Glucose Activates ROS-NLRP3 Inflammasome Signaling in GMCs

**Figure 1:**
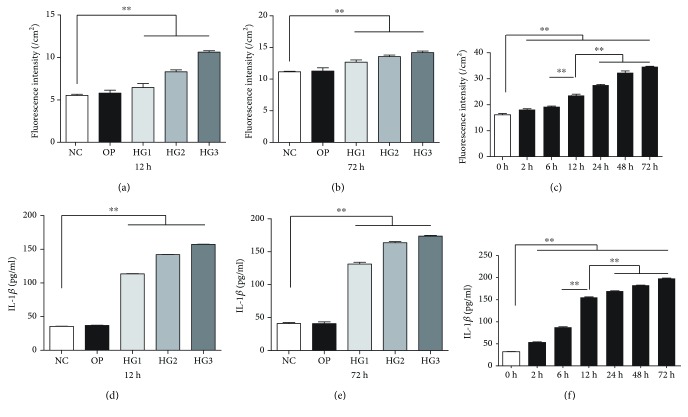
Elevated ROS and IL-1*β* in SVs after HG exposure. HG enhanced ROS production compared to the NC and OP groups when cultured for 12 h (a) and 72 h (b). HG also induced a time-dependent increase in ROS production (c). The level of IL-1*β* increased in the HG group for 12 h (d) and 72 h (e), evaluated by ELISA. Moreover, HG caused an increase in IL-1*β* in a time-dependent manner (f). ^∗∗^*p* < 0.01.

**Figure 2:**
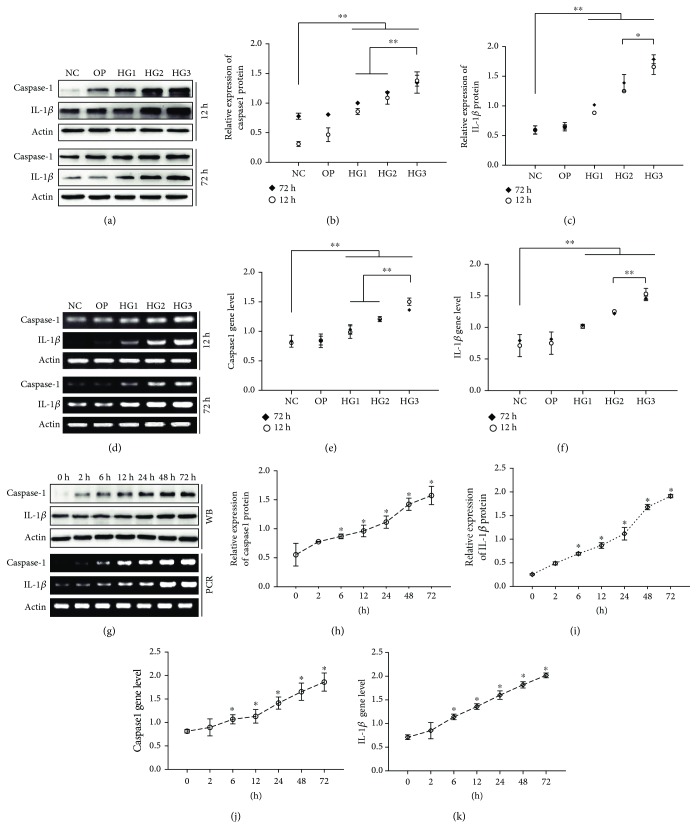
HG activated NLRP3 inflammasome signaling in SVs. Immunoblots (a–c) and RT-PCR (d–f) showing caspase1 and IL-1*β* expression in each group. Caspase1 and IL-1*β* increased in the HG group in a concentration-dependent manner at both 12 h and 72 h, especially for 72 h. HG also caused an increase in caspase1 and IL-1*β* in a time-dependent manner detected by western blotting and RT-PCR (g–k). ^∗∗^*p* < 0.01; ^∗^*p* < 0.05.

High glucose is the main driving force of DN development, while oxidative stress and inflammation are considered as key molecular mechanisms involved in the pathophysiology of DN [26]. In this study, we assessed the levels of ROS and the NLRP3 inflammasome (identified by caspase1 and IL-1*β*); DCFH assays were applied to examine whole ROS production. Results showed that total ROS production was significantly increased in the HG group compared with the NC group and the OP group, in a dose- and time-dependent manner (*p* < 0.01) (Figures 1(a)–1(c)). ELISA, western blotting, and RT-PCR (Figure 2) were used to identify the expression of caspase1 and IL-1*β*. Compared with the NC group and the OP group, the expression of caspase1 and IL-1*β* increased significantly in the HG group with a dose- and time-dependent manner (*p* < 0.01), especially for the HG3 group, while there were no significant differences between the NC and OP groups regarding levels of ROS, caspase1, and IL-1*β* (*p* > 0.05).

### 3.2. Autophagy Negatively Regulates ROS-NLRP3 Inflammasome Signaling in GMCs Stimulated by High Glucose

**Figure 3:**
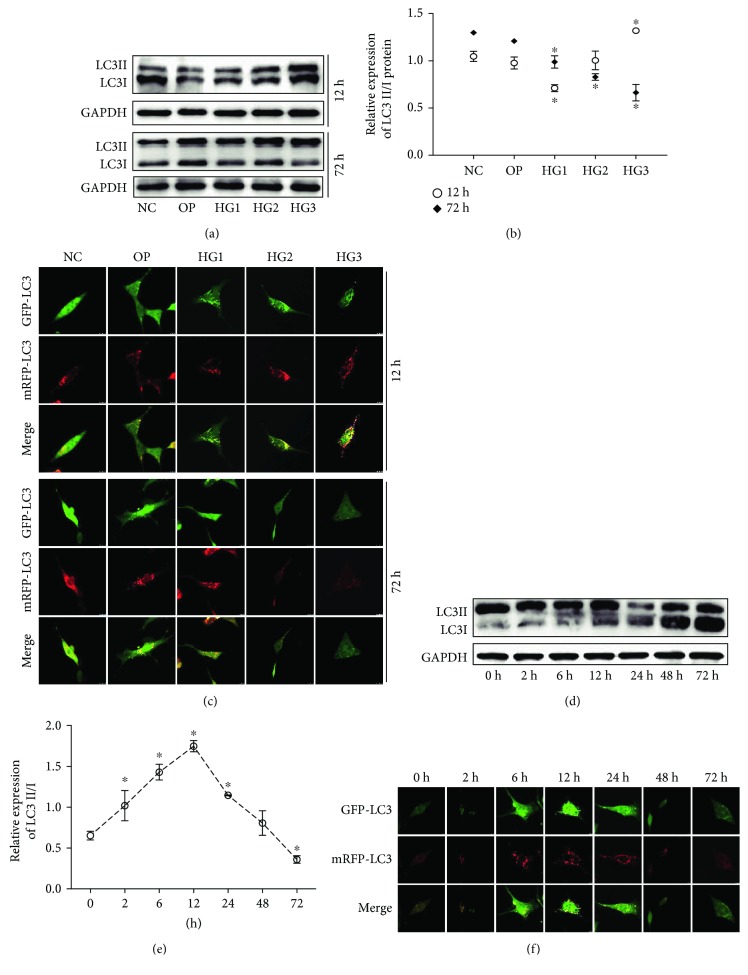
LC3 expression in HG-induced SV cells. LC3II/I expression increased in the HG group in a concentration-dependent manner at both 12 h and 72 h, especially for 72 h (a, b). In addition, HG caused an increase in LC3II/I in a time-dependent manner, detected by western blotting (d, e). Representative confocal microscopy images of tfLC3 puncta in SVs stained for autophagosomes (green) and autolysosomes (red) for colocalization as an indicator of autophagy. We observed that autophagy increased in the HG group in a concentration-dependent and time-dependent manner (c, f). ^∗^*p* < 0.05.

**Figure 4:**
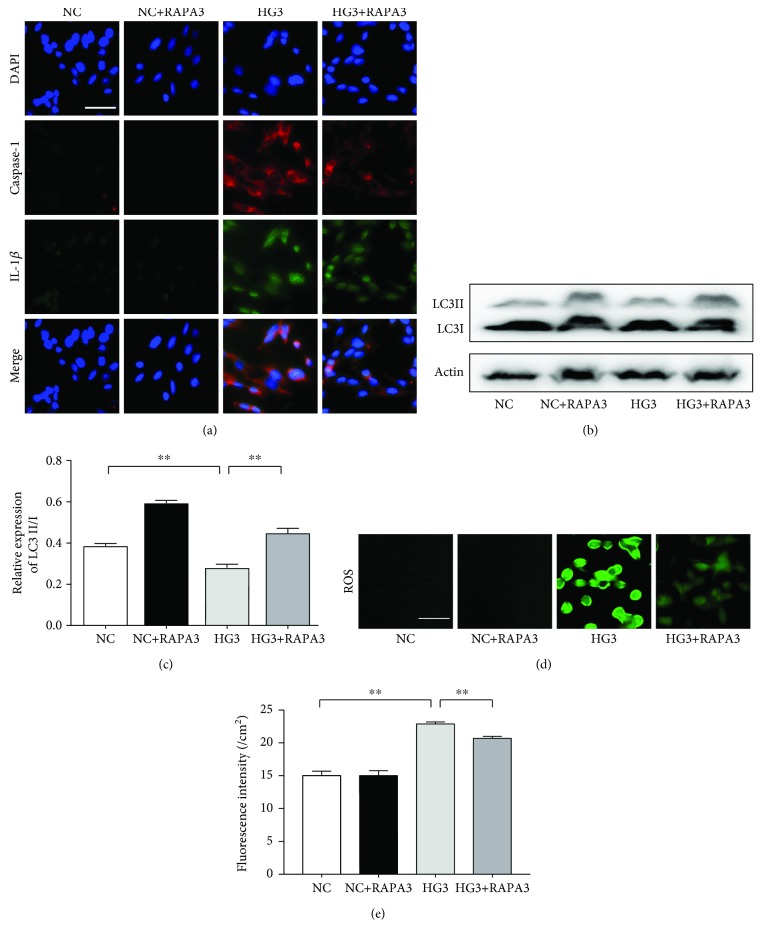
Effects of rapamycin on autophagy and ROS-NLRP3 inflammasome signaling in renal mesangial cells. (a) Caspase1 and IL-1*β* detected by immunofluorescence. (b) LC3 detected by western blot. (c) The corresponding relative gray value statistics graph of LC3B. (d, e) ROS. LC3 expression increased following rapamycin treatment compared with the NC and HG3 groups (*p* < 0.01), while the level of ROS, caspase1, and IL-1*β* was decreased (*p* < 0.01).

In many investigations, autophagy has been reported to negatively regulate activation of the inflammasome by elevating the damage in mitochondria and recycling intracellular energy resources; autophagy can also decrease ROS levels and inhibit the activation of the NLRP3 inflammasome induced by ROS [27–29]. In our study, we used the LC3-GFP autophagic puncta and the LC3II/I ratio (marker of autophagy) to test the level of autophagy after exposure to HG. Western blot analysis was used to examine the LC3II/I ratio, and confocal microscopy was used to assess the mean number of autophagosomes (yellow) or autolysosomes (red). We detected a high basal level of autophagy in the NC group and a low level of autophagy in the HG1 and HG2 groups; however, there was a concentration-dependent increase in autophagy in the HG groups at 12 h, especially in the HG3 group (*p* < 0.01) (Figures 3(a)–3(c)), contrary to the popular view that high glucose inhibits autophagy in GMCs [29–31] (although these experiments were carried out at 48 h of HG or more). We therefore also observed the autophagy level for 72 h at different concentrations of HG, and we found a decrease in LC3 expression in the HG group compared with the NC group; the lowest LC3 expression was found in the HG3 group (*p* < 0.01) (Figures 3(a)–3(c)). Thus, we hypothesized that autophagy levels were closely related to the culture times of HG and that HG enhanced autophagy during short periods but inhibited autophagy in the long term. To confirm this, we examined the level of autophagy in GMCs in the HG3 groups for different times (0 h, 2 h, 6 h, 12 h, 24 h, 48 h, and 72 h), and our data confirmed a short term-dependent increase in autophagy in the HG3 group, which reached a peak at 12 h (*p* < 0.01) (Figures 3(d)–3(f)). However, the number of autophagosomes and LC3 levels decreased in the HG groups at 72 h compared with the NC and OP groups and demonstrated a concentration-dependent manner in HG groups (*p* < 0.05, Figures 3(d)–3(f)). At the same time, ROS, caspase1, and IL-1*β* increase with a time- and dose-dependent manner in the HG group (*p* < 0.05), which prompted that autophagy may negatively regulate ROS-NLRP3 inflammasome signaling in GMCs stimulated by high glucose. To test the hypothesis, we used rapamycin as a specific activator of autophagy to interfere with the cells for 72 hours and tested the protein expression of LC3II/I, ROS, caspase1, and IL-1*β* in NC, NC+rapamycin (10 nmol/L), HG3, and HG3+rapamycin (10 nmol/L) groups. And we found that LC3 expression increased following rapamycin treatment compared with the NC and HG3 groups (*p* < 0.01, Figures 4(b) and 4(c)), while the level of ROS, caspase1, and IL-1*β* was decreased (*p* < 0.01, Figures 4(a), 4(d), and 4(e)).

### 3.3. RIPK2 Regulates the NLRP3 Inflammasome through Autophagy

**Figure 5:**
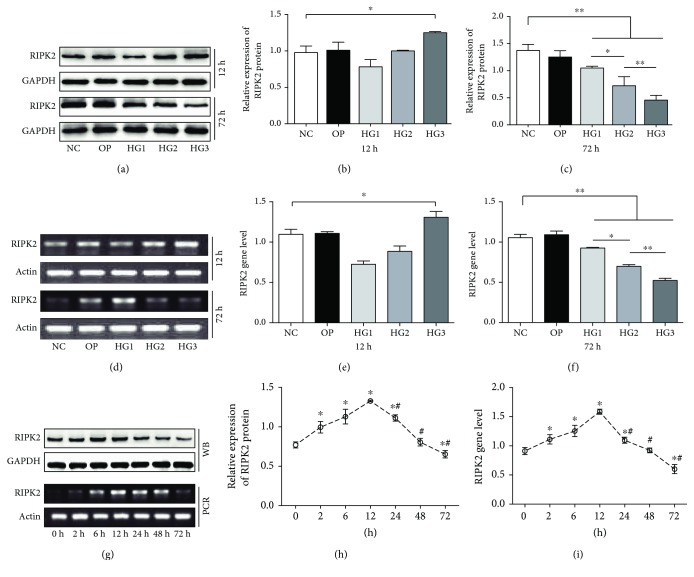
RIPK2 expression in HG-induced SV cells. (a–f) RIPK2 increased in the HG group both at 12 h and at 72 h, detected by western blotting and RT-PCR. HG caused an increase in RIPK2 at 2–12 h, reached a peak at 12 h, and then reduced in a time-dependent manner (g–i).

**Figure 6:**
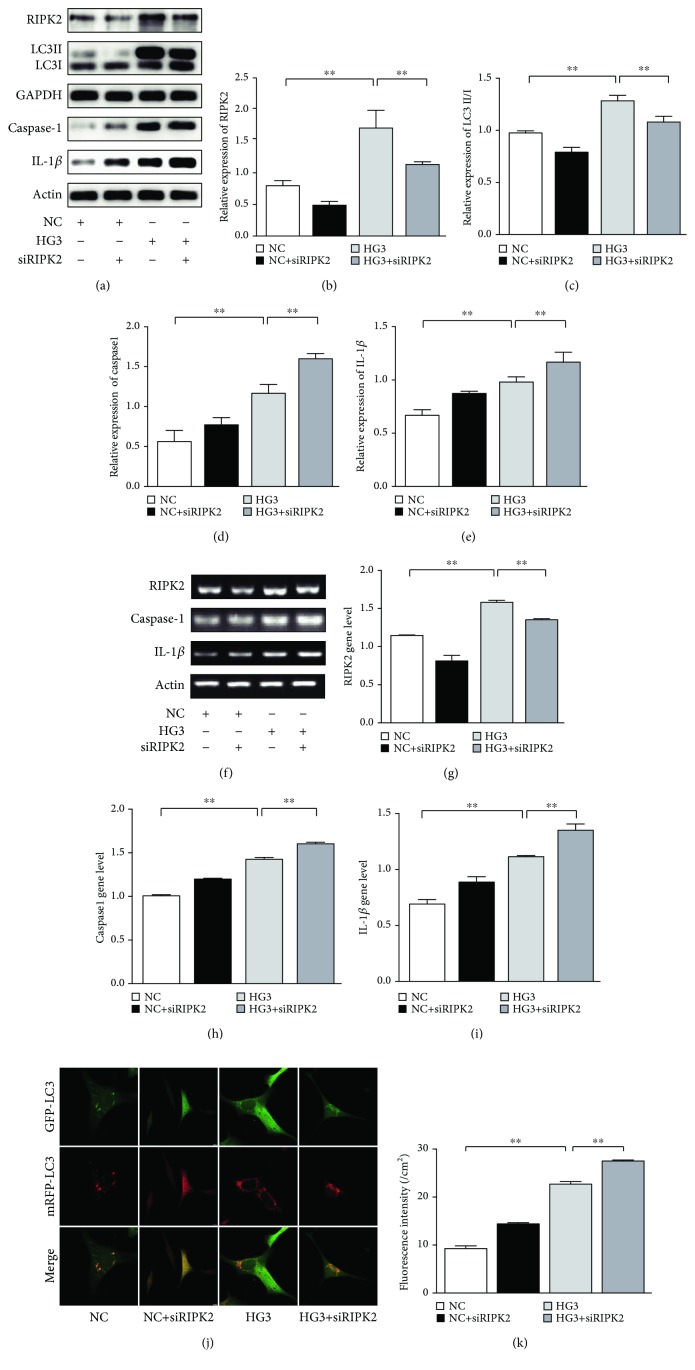
RIPK2 regulates the NLRP3 inflammasome through autophagy. (a) RIPK2, LC3B, caspase1, and IL-1*β* in SVs after siRIPK2 for 12 h were detected by WB. (b-e) The statistical chart of relative gray value of WB band. (f) RIPK2, caspase1, and IL-1*β* in SVs after siRIPK2 for 12 h were detected by RT-PCR; (g–i) the statistical chart of relative gray value of RT-PCR band. We detected that LC3B was decreased with RIPK2, but caspase1 and IL-1*β* were increased. Representative confocal microscopy images of tfLC3 puncta in siRIPK2 groups compared to the NC and HG3 groups for 12 h (i); we observed that autophagy increased in the HG group and was inhibited after siRIPK2, but the ROS production was increased in the HG group after siRIPK2 for 12 h (h). ^∗∗^*p* < 0.01.

The receptor-interacting protein kinase 2 (RIPK2) is a serine/threonine kinase with a carboxy-terminal caspase activation and recruitment domain (CARD); it is important for activating the NF-*κ*B- and MAPK-dependent inflammatory responses [32, 33]; in recent investigations, it was shown that RIPK2 regulates autophagy and inflammasome activation [18–20]. However, there has been no research into the expression of RIPK2 and its effects on autophagy in renal mesangial cells cultured in HG. Thus, we first assessed the expression of RIPK2 in SVs stimulated by HG, and we found that HG could increase RIPK2 in a concentration-dependent manner up to 12 h, which reached a peak in the HG3 group (*p* < 0.01), and a statistical difference (*p* < 0.01) was observed between HG group comparisons (Figures 5(a)–5(c)), while HG3 resulted in decreased RIPK2 expression in a concentration-dependent manner up to 72 h (Figures 5(d)–5(f)), and a statistical difference (*p* < 0.01) was also observed between HG group comparisons. We then silenced the RIPK2 gene by siRNA transfection and found that RIPK2 and LC3 expression decreased following siRIPK2 treatment compared with the HG3 group at 12 h, while the level of ROS, caspase1, and IL-1*β* was enhanced in the siRIPK2 group (Figure 6).

## 4. Discussion

Among the multifactor mechanisms of the hyperglycemic effects on renal injury, oxidative reactions and inflammation were shown to act as triggers, modulators, and links within the complex web of pathological events that occur in DN [34–36]. Hyperglycemia induces the oxidative reaction and releases ROS, as well as activating the subsequent NLRP3 inflammasome signaling, resulting in injury of podocytes or tubular cells and mesangial cell proliferation, leading to the occurrence and development of DN [3]. Our study was conducted to explore the potential role and mechanisms of RIPK2 and autophagy and their relationship with ROS-NLRP3 inflammasome signaling of DN. The results demonstrated enhanced ROS-NLRP3 inflammasome signaling and increased formation of autophagosomes in GMCs under HG conditions for a short period. Furthermore, silencing of the RIP2 gene, using siRNA technology, reduced the formation of autophagosomes in GMCs exposed to HG.

### 4.1. Autophagy Negatively Regulates HG-Induced ROS-NLRP3 Inflammasome Signaling

Recent studies have reported that defective autophagy in podocytes and tubular cells facilitated HG-induced renal injury in cells and in DN models. Under normal circumstances, podocytes maintain a high level of autophagy, and renal tubular cells maintain a low level of autophagy in diabetes or DN models [37–39]. Almost all studies indicate renoprotection by autophagy of podocytes, while the effects of autophagy for renal tubular cells in diabetes or DN models are controversial. Tagawa et al. [40] found defective autophagy and podocyte apoptosis in DN patients and mice with proteinuria; specifically, Atg5 knockout can accelerate diabetes-induced podocyte injury, filtration barrier destruction, and glomerular sclerosis [8]. Most studies have demonstrated that autophagy in renal tubular cells was inhibited in diabetic conditions and suggested a renoprotective role when autophagy was enhanced, while several investigations found increased autophagy in renal tubular cells of DN that was, in fact, harmful to the kidney. We found that these studies examined formation of autophagosomes without detecting autophagy-lysosomal degradation, which is not enough to represent autophagy activity. Huang et al. [41] showed that LC3 and p62 expression in tubular epithelial cells increased significantly and found that HG may enhance autophagosome formation while inhibiting autophagy-lysosomal degradation; studies on autophagy activity of GMCs are, however, very limited, and effects of short- and long-term HG on autophagy in GMCs have also not been investigated. Therefore, based on the limited results, HG has been recognized to have the effect of inhibiting autophagy of GMCs, which would accelerate the development of kidney diseases.

The most interesting finding of this study is that short-term exposure to hyperglycemia can increase autophagy of GMCs, which is contrary to the traditional concept of autophagy. In fact, we observed that autophagy negatively regulates HG-induced ROS-NLRP3 inflammasome signaling, while HG has a dual role on autophagy. Firstly, HG enhanced autophagy in the short term (0-12 h) in a dose-dependent manner and negatively regulated ROS-NLRP3 inflammasome signaling, while continuous and chronic hyperglycemia actually inhibited the level of autophagy, resulting in increased expression of ROS, caspase1, and IL-1*β*. This indicates that HG has different effects on autophagy in renal mesangial cells, according to the time of HG exposure. In addition, as expected, autophagy negatively regulates ROS-NLRP3 inflammasome signaling. Moreover, our study also found that HG increased ROS-NLRP3 inflammasome in a time- and dose-dependent manner, with enhanced autophagy. It has been estimated that ROS-induced cellular oxidative stress is a major modulator of autophagy. However, autophagy is also able to regulate ROS formation [42, 43]; ROS increased with accumulation of damaged mitochondria when cells were exposed to HG, and this may induce autophagy by inhibiting mTOR and upregulating ERK or JNK p53 [44, 45]. The increased autophagy can reverse the damaged mitochondria and isolate the NLRP3-binding protein ASC to prevent caspase1-mediated pro-IL-1*β* decomposition into the active form, reduce ROS accumulation, and inhibit the activation of inflammation signals of NLRP3. When a balance was reached between autophagy and ROS, there was a peak in autophagy. However, if hyperglycemia was not rectified, the accumulation of damaged mitochondria, other ubiquitin, and injured organelles exceeds the autophagy-scavenging capacity, thus releasing ROS and mitochondrial DNA into the cytoplasm, causing continuous activation of NLRP3 inflammasome, which indicates that excessive intracellular stress resulting from hyperglycemia may lead to autophagy as a consequence of cellular damage.

### 4.2. RIPK2 Regulates the NLRP3 Inflammasome through Autophagy

RIPK2 has been estimated to be an essential component in NOD1/NOD 2-dependent autophagy despite its role in the activation of NF-*κ*B, p38 MAPK, JNK, and ERK1/2 [12–16]. Previous studies have shown that blocking RIPK2 signaling could decrease autophagosome formation [46], and RIPK2 kinase activity may play a dual role in NOD-dependent autophagy [21]. Our research has shown that HG activates RIPK2 in the short term (0-12 h) and inhibits RIPK2 in the long term, which is consistent with the trend of LC3 expression. This indicates that RIPK2 may have a potential role in mediating autophagy, which was identified in our silenced group that was transfected with siRNA-targeting RIPK2. Several possible mechanisms may be involved in the mediation of RIPK2 on autophagy, as mentioned above, autophagy is regulated by nutrient-responsive intracellular signals such as mTORC1, AMPK, and Sirt1; the key proteins in these signaling pathways converge together at the NOD-RIPK2-NF-*κ*B/p38 MAPK signal. Thus, we hypothesized that HG induced RIPK2-dependent NF-*κ*B signal, causing the release of ROS and ATP depletion, which sends a positive autophagy signal by p38 MAPK activation, as well as directly activating ULK1 and inhibiting the suppressive effect of MTORC1 complex1 on ULK1, which has been shown in a previous study [47]. While Lupfer et al. [46] indicated that RIPK2 regulates autophagy through its kinase activity instead of p38 MAPK, which had a lower level of Ser555 phosphorylation in ULK1 in RIPK2^−/−^ BMDCs during IAV infection, upregulation of total ULK1 was not affected. Therefore, p38 MAPK has been reported to either negatively or positively regulate autophagy dependent on stimulus as well as cell context, and whether autophagy is stimulated by p38-dependent ROS generation or activation of p38 by ROS needs further investigation.

Our study also observed a decrease of RIPK2 and LC3 when SVs were exposed to HG for 12 h or more. Previous studies have indicated that RIPK2 is related to innate immunity, inflammation, and autophagy and is involved in atherosclerosis, bacterial or viral infection, Crohn's disease, and so on [20, 46, 48–50]. Several studies investigated the RIPK2 inhibitor, for example, the use of SB203580 and WEHI-345 [46, 51]. More recently, increasing evidence has suggested an alternative mechanism of autophagy activation that requires RIPK2 [46, 47]. Thus, we suggest that there was a RIPK2-dependent regulated mechanism of autophagy in HG-cultured GMCs and the possible mechanism. Other limited research has shown that PP2A, a ubiquitous serine/threonine phosphatase, has been shown to activate p38 as well as other MAPKs [21] and ATG16L1 and could negatively regulate the activation of RIPK2 and autophagy, avoiding excessive autophagy [52].

In this study, we studied the role of high glucose in the regulation of autophagy in renal mesangial cells *in vitro*. GMCs are the main target of renal inflammation and fibrosis. And GMCs stimulated by high glucose can simulate well the occurrence and development of diabetic nephropathy. However, our study also has limitation, we did not study on kidney biopsies from patients with diabetic nephropathy or on animal models. And renal biopsy for detecting RIPK2, autophagy, and ROS-NLRP3 levels in diabetic patients without renal damage and diabetic nephropathy patients with different classes of renal damage may provide new basis for early diagnosis and new target for advanced treatment in diabetic nephropathy. Therefore, further studies about RIPK2, autophagy, and ROS-NLRP3 signal *in vivo* need to be continued.

**Figure 7:**
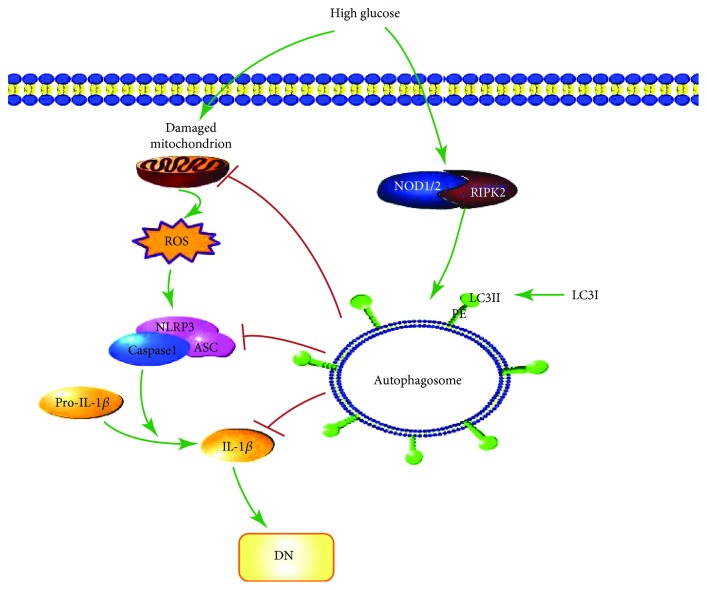
Diagram of the RIPK2-induced regulation of ROS/NLRP3 inflammasome by autophagy.

In conclusion, we have demonstrated that high glucose plays a dual role in autophagy in GMCs, can activate the autophagy signaling pathway in short time periods, and can inhibit autophagy over 12 h. Autophagy negatively regulates ROS-NLRP3 inflammasome signaling induced by high glucose and is involved in the pathogenesis of DN. In addition, RIPK2 may play a vital role in autophagy. This finding suggests that the activation of RIPK to induce autophagy of GMCs may be a potential therapeutic strategy of DN (Figure 7) and further studies *in vivo* are needed.

## Data Availability

The data used to support the findings of this study are included within the article.
